# An Ergonomics Analysis of Archers through Motion Tracking to Prevent Injuries and Improve Performance

**DOI:** 10.3390/s24061862

**Published:** 2024-03-14

**Authors:** Xiaoxu Ji, Jenna Miller, Xin Gao, Zainab Al Tamimi, Irati Arzalluz, Davide Piovesan

**Affiliations:** 1 Biomedical Engineering, Gannon University, Erie, PA 16541, USA; miller311@gannon.edu (J.M.); altamimi005@gannon.edu (Z.A.T.); arzalluz001@gannon.edu (I.A.); piovesan001@gannon.edu (D.P.); 2 Electrical and Computer Engineering, Gannon University, Erie, PA 16541, USA; gao005@gannon.edu

**Keywords:** archery, motion tracking, ergonomics, Xsens, JACK Siemens, injury, lower back

## Abstract

Archery ranks among the sports with a high incidence of upper extremity injuries, particularly affecting the drawing shoulder and elbow, as well as inducing stress on the lower back. This study seeks to bridge the gap by integrating real-time human motion with biomechanical software to enhance the ergonomics of archers. Thirteen participants were involved in four tasks, using different bows with varied draw weights and shooting distances. Through the application of advanced integrative technology, this study highlights the distinct postures adopted by both males and females, which indicate the biomechanical differences between genders. Additionally, an analysis of the correlation between exposed spinal forces and these adopted postures provides insights into injury risk assessment during the key archery movements. The findings of this study have the potential to significantly enhance the application of training methodologies and the design of assistive devices. These improvements are geared towards mitigating injury risks and enhancing the overall performance of archers.

## 1. Introduction

Archery, a versatile activity with origins dating back to 20,000 B.C., has played significant roles in hunting, warfare, recreation, and sports [1]. Since its inclusion in the Olympic Games in 1900 [1], archery has experienced a surge in global popularity, captivating millions of enthusiasts [2]. In the 2021–2022 season alone, the number of actively licensed bowhunters reached 3.7 million [3]. In the United States, approximately 21.6 million adults, comprising 9.2% of the population, participated in archery in 2014 [4].

Beyond its recreational appeal, archery holds significant value in healthcare and education. It serves as a highly effective therapeutic tool for individuals facing physical disabilities, such as those with high spinal cord injuries (SCIs) [5,6]. Moreover, archery is seamlessly integrated into the Expanded Core Curriculum, providing valuable benefits to schoolchildren with deaf-blindness or visual impairment [7]. A noteworthy report indicates that pediatric archers, on average, surpass both athletes and non-athletes in basketball, football, and swimming in terms of overall pulmonary functions and respiratory muscle strength [8]. Consequently, archery has emerged not only as a historic and recreational pursuit, but also as a multifaceted activity with diverse benefits spanning education, health, and conservation realms.

Recognized as an exceptionally versatile sport, archery is esteemed for its recreational and rehabilitative attributes; however, approximately 45–50% of archers experience shoulder-related injuries during their athletic careers [9]. In total, 83.4% of injuries occurring in the sport of archery result in the upper extremities [10]. One out of two people who suffer from an upper extremity musculoskeletal injury are unable to continue to practice archery [11]. Archery, ranking alongside baseball, is the highest-ranking sport exposed to upper extremity injuries [11]. Among the upper extremities, the majority of reported injuries occur in the shoulder and back, particularly in the drawing shoulder and elbow of the archer [12]. Shoulder instability has been identified as a key underlying cause of many upper extremity injuries in archery. According to Taylor Kuhlmann, the repetitive action of lifting and drawing back the bow can lead to scapular dyskinesis, torn rotator cuffs, and bursitis, all stemming from shoulder instability [13]. Continuous, identical movements of the drawing shoulder under a constant load contribute to shoulder instability and impingement fatigue [14]. Furthermore, repetitive reloading of the spine leads to a cumulative stress buildup, resulting in early degenerative changes [15]. In the first quarter of 2021, over 2000 archers became members of USA Archery, indicating the growing popularity of the sport nationwide [16]. Consequently, there is an increased concern for the health and safety of both current and future athletes.

Various techniques have been explored to mitigate injury risks in archers, such as stretches, the establishment of a muscle fatigue benchmark, strengthening exercises, and biofeedback. The prevention of muscle fatigue involves identifying the muscle force at each archery stage and comparing it to a benchmark force generated by a professional archer [17]. Plyometric training has demonstrated the enhanced performance and accuracy among archers [18]. Biofeedback contributes to concentration, subsequently improving frequency levels [19]. Additionally, finite element modeling (FEM) has been instrumental in preventing musculoskeletal injuries in archery. Simulations using a finite element model of a drawing side shoulder joint analyzed stress changes on the upper extremities. Electromyography, coupled with musculoskeletal simulation, has examined the shoulder joint within biomechanical models [20]. However, limitations exist in prior research. While biofeedback devices enhance concentration and frequency levels, they may fall short in addressing shoulder instability over the long term. FEM relies on approximations during model construction, introducing potential errors. Biomechanical models do not always anticipate human movement and errors during task performance.

Currently, numerous studies are underway to assess techniques related to ergonomics and force variations among archers. The majority of the techniques are survey-based, FEM-based, or model-based [21]. However, there is a limited emphasis on kinematic approaches as the primary mode of data collection. Additionally, there is an absence of studies that present data by simulating the shooting process using computer models or utilizing ergonomic software for motion data optimization. Thus, this study aims to bridge this gap by employing real-time motion tracking. Xsens motion tracking sensors were placed on the body to record subjects in real-time motion. The Xsens setup is portable, offering convenient data collection with a straightforward process. This method is not constrained by area recording or light conditions [22]. Moreover, it proves effective in correcting stance, posture, and movement [23]. The recorded data were then processed using JACK Siemens software (v9.0) (Plano, TX, USA) to improve the ergonomics of archers. Through the integration of real-time motion trackers and human modeling simulations, the collected data for each subject will provide insights into the injury risk assessment during the key archery movement—holding up the bow and arrow, drawing the bow, and the release of maximum force from the subject’s body. The main aim of this study is to employ human modeling simulation, enabling the real-time analysis of joints and forces in the upper extremities and back for each participant. This advanced technique utilizes a technological combination to improve shoulder and elbow joint mobility in archers, subsequently reducing the risk of lower back injuries by minimizing strain and stress on each joint. The ultimate goal is injury prevention through enhanced biomechanical understanding.

## 2. Methods

### 2.1. Participants and Software

This experimental study was conducted in accordance with the Declaration of Helsinki and was approved by the Gannon Institutional Review Board (GUIRB-2023-3-7063) for studies involving humans. Participation was completely voluntary. For eligibility in the study, participants had to be in good health without any conditions that could impede the movement of their legs or arms. Clear explanations about the study’s objectives were provided to the participants, and their information was assessed to ascertain their compliance with the inclusion criteria. In total, thirteen college students were recruited, most through the archery club, which also supplied the shooting range and appropriate equipment to carry out the necessary tasks.

This study enrolled thirteen participants (6 females and 7 males), with an average body height and body weight (reported as the mean ± standard deviation) of 162.5 ± 7.5 cm and 66.2 ± 17.2 kg for females and 174.7 ± 9.6 cm and 75.4 ± 20.4 kg for males, with ages ranging from 18 to 30 as shown in Table 1. Additionally, measurements of upper and lower limb lengths, shoulder width, hip width, and arm span were taken to facilitate the creation of individual digital human models (DHMs) for each participant.

Data collection of live motion tracking of the archers’ movements was performed using the Xsens MVN Awinda system (Enschede, The Netherlands) [24]. The 17 inertial-based sensors were secured on the body segments, such as the head, sternum, shoulders, upper arms, forearms, hands, pelvis, thighs, shanks, and feet. The first DHM of each participant was generated in MVN Analyze software (DHM_Xsens). The integration of accelerometers, gyroscopes, and magnetometers within a unified inertial sensor providing acceleration input through a Kalman filter yielded Xsens resistance against orientation drift. This advanced technology empowers Xsens to precisely estimate micro-movements, facilitating the subtle differentiation between individuals in precision-demanding shooting sports such as archery [23,25]. A second DHM was developed in JACK Siemens PLM software (DHM_JACK) [26] for injury risk analysis.

**Figure 1:**
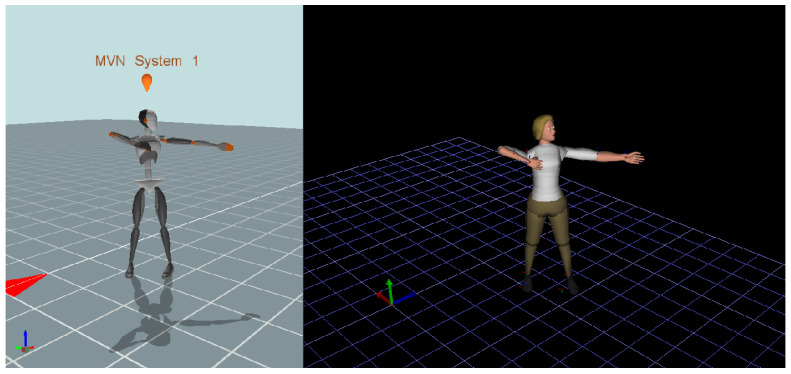
Synchronous movements from Xsens software (v2019) (**left**) and JACK software (v9.0) (**right**).

The integration of both DHMs, which were developed based on anthropometric data gathered from participants during the orientation session, facilitates a seamless blend for precise human motion simulation. The alignment of skeletal segments from DHM_Xsens with human body joints from DHM_JACK was achieved by correctly transferring data through a designated port number in the network streamer, especially during the N-pose of the mannequin in the Xsens recording. This unique feature, harnessing both Xsens and JACK, is illustrated in Figure 1, where DHM_Xsens is presented on the left side and the corresponding DHM_JACK is on the right side. The figure illustrates the replication of real human movement.

### 2.2. Operational Tasks

**Figure 2:**
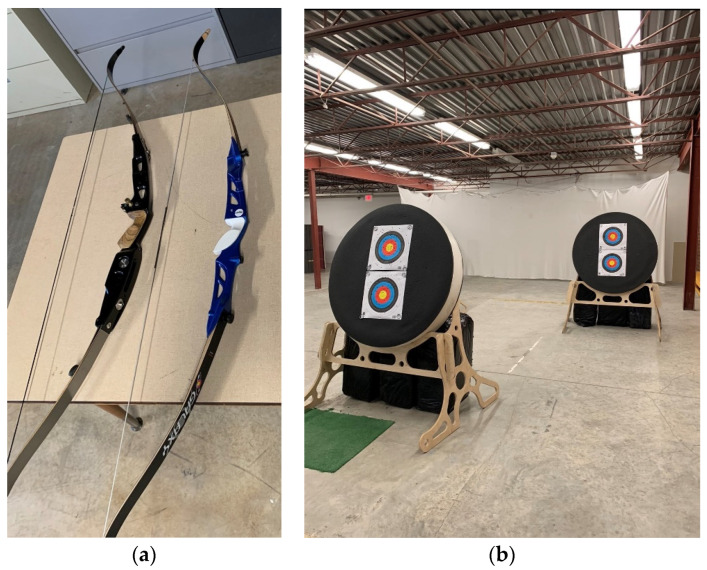
(**a**) Bows with different draw weights: 25 lbs (blue) and 32 lbs (black). (**b**) Targets at two different distances.

A combination comprises 5 arrow shots, typically executed in under a minute, targeting specific parameters such as target height, distance, and bow poundage. Each participant was required to perform four tasks, involving the use of two bows with draw weights of 25 lbs and 32 lbs at two distances (10 yards and 15 yards), as depicted in Figure 2. The net weights of the respective bows were 11 kg and 15 kg. The distance from the center of the bottom target to the floor was set at 100 cm. Each archer participant was tasked to shoot each target with 5 arrows with both bows, making a total of 20 unique shots. The four tasks are listed below.

Task#1: Pulled the bow with a draw weight of 25 lbs and aimed at a close distance (10 yards).

Task#2: Pulled the bow with a draw weight of 25 lbs and aimed at a far distance (15 yards).

Task#3: Pulled the bow with a draw weight of 32 lbs and aimed at a close distance (10 yards).

Task#4: Pulled the bow with a draw weight of 32 lbs and aimed at a far distance (15 yards).

Our study was conducted following USA Archery guidelines, utilizing the recurve bow type and adhering to the target form of archery. Each participant underwent guidance through the shooting sequence, aiming to achieve their best performance. Prior to the commencement of the study and each bow switch, participants were afforded the opportunity to engage in a warm-up session until they felt adequately prepared. The range was supervised by a certified archery coach, who provided instructions on the safe and methodical execution of the shooting procedure beforehand.

Each participant executed a sequence of movements to achieve a successful shot. First, they positioned their bodies directly facing the target with feet shoulder-width apart. Then, arrows were positioned on the bowstring and secured onto the arrow rest. Subsequently, participants pulled the bowstring towards their faces, with shoulders slightly rotating inward. Throughout this process, each participant kept their elbow close to the body, ensuring that the bow arm extended straight out. The bowstring was released after aligning the sight on the bow with the target.

### 2.3. Data Analysis

**Figure 3:**
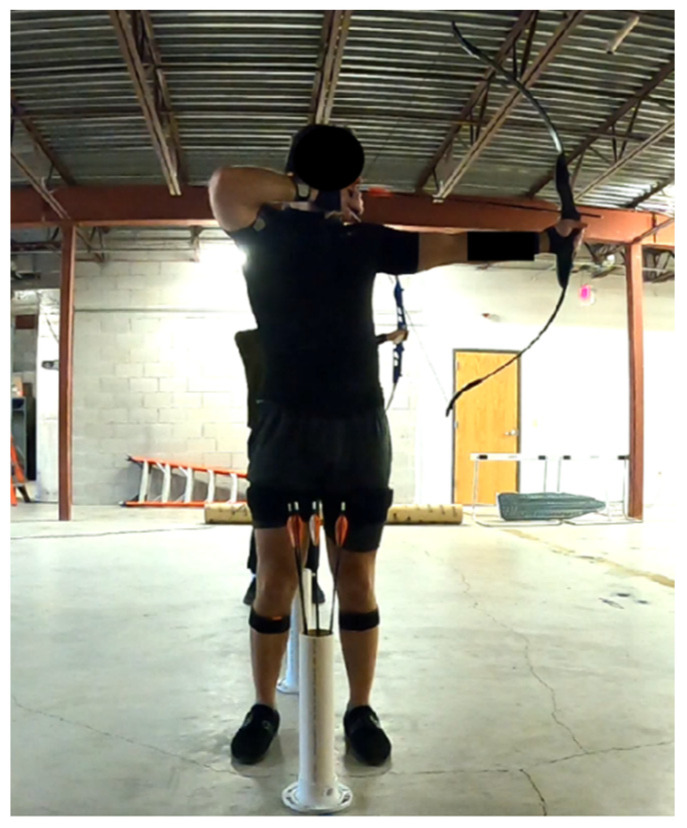
The identified specific pose that may cause injury to the lower back.

The chosen pose for each combination occurred just as the participant was about to release the bowstring to propel the arrow. This specific posture, depicted in Figure 3, was identified as a potential source of injury risk to the lower back (4th/5th lumbar spine) and shoulder for each participant. This heightened risk is attributed to the substantial force applied on the bow during the shooting sequence through both hands, resulting in the largest reaction forces on the body through the same contact points from the bow.

The force exerted by both hands was quantified using a digital force gauge (SF-500). By merging the adopted posture with the applied hand force, a comprehensive analysis of compressive force and the associated joint angles—encompassing shoulders, elbows, and trunk—was conducted, allowing for gender- and task-specific assessments.

To assess the spinal forces exerted, we initiated the process by determining the external reaction forces acting on the body. The draw hand, being the dominant hand responsible for pulling and holding the bowstring, experiences an overall horizontal compressive force at the index knuckle. Simultaneously, the bow hand, responsible for gripping, supporting the bow, and maintaining a forward position as the string is drawn and held back, encounters an extra vertical downward force at the palm center. The JACK Siemens’ Force Solver was utilized to identify specific points of application for these contact forces.

The force applied to the draw hand is considered purely horizontal, resulting in x-values (anterior–posterior) and z-values (lateral–medial), while the y-value (superior–inferior) was negligible. The x- and z-values, ranging from −1.0 to 1.0, were estimated by aligning the arrow vector in JACK software parallel to the virtual arrow and the compressive horizontal component on the bow hand. Conversely, the bow hand experiences two reaction forces, leading to the emergence of a resultant force. The x- and z-values for the bow hand should be of equal but opposite magnitudes to those selected for the draw hand, while the y-value, representing a downward force, needed to be calculated. Considering that the resultant vector of x- and z-directions was consistently close to 1.0, Equation (1) could be utilized to determine the y-value (Vy) within the specified coordinate system.

(1) $$\frac{V_{xz}}{V_{y}}=\frac{F_{xz}}{F_{y}}$$

where $V_{xz}$ is the resultant vector of the x- and z-directions, $V_{y}$ is the vector y-value within the given coordinate system, $F_{xz}$ is the measured horizontal force using the digital force gauge (SF-500), and $F_{y}$ is the weight of bow.

In an optimal scenario, the arrows should exhibit a slight superimposition in their respective arms. These arrows create a connection between the two hands through a line of action that aligns with the longitudinal axis of the arrow shaft. Consequently, the compressive forces exerted on the 4th and 5th lumbar vertebrae (L4/L5) for each subject in this particular pose were estimated using JACK software.

The joint angles pertinent to this study are the abduction/adduction, internal/external rotation, and flexion/extension of the right and left shoulders, the flexion/extension of the right and left elbows, and the flexion/extension of trunk. These provide a measure of consistency between shots that helps relate the positioning of shooting performance to injury risk.

### 2.4. Statistical Analysis

For each pose, a two-way analysis of variance (ANOVA) was conducted to analyze the variables of spinal force and anatomical joints concerning genders and tasks. To obtain a thorough comprehension of each variable, we conducted a *t*-test to examine variations in compressive force and joint angles across different genders and tasks as well. A significant level of 0.05 was established. Furthermore, a cross-correlation value was computed between the exerted spinal force and the primary anthropometric variables to assess their influence on the risk of lower back injury.

## 3. Results

Table 2 presents the exposed spinal forces and joint angles. The term “Comp” denotes compressive force exerted on the lower back, while “R_Shoulder” and “L_Shoulder” represent the right and left shoulders, respectively. Similarly, “R_Elbow” and “L_Elbow” indicate the right and left elbows. “F/E” stands for flexion and extension. “Abd/Add” stands for abduction/adduction, and “Rot” stands for rotation. “AVE_F” and “AVE_M” represent average values for females and males. Additionally, “25C”, “25F”, “32C”, and “32F” are used to designate bows with 25 lb and 32 lb draw weights aimed at close and far distances, respectively.

**Figure 4:**
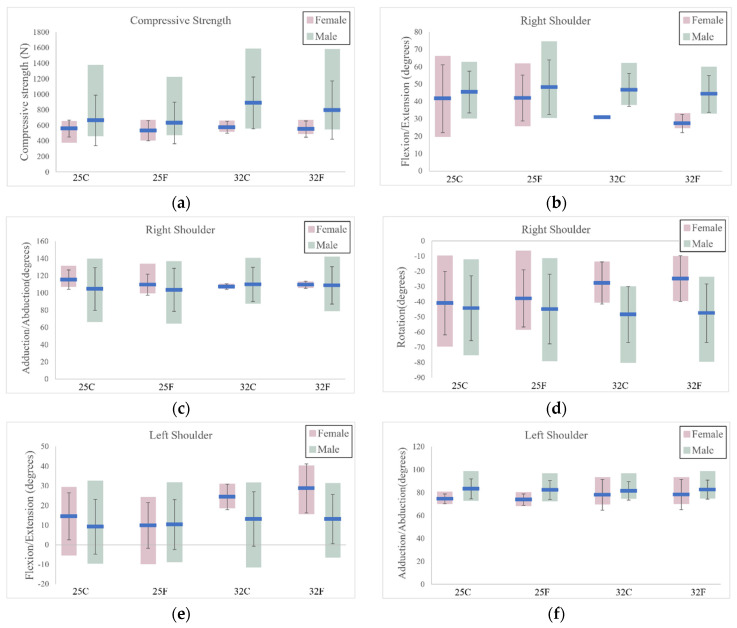

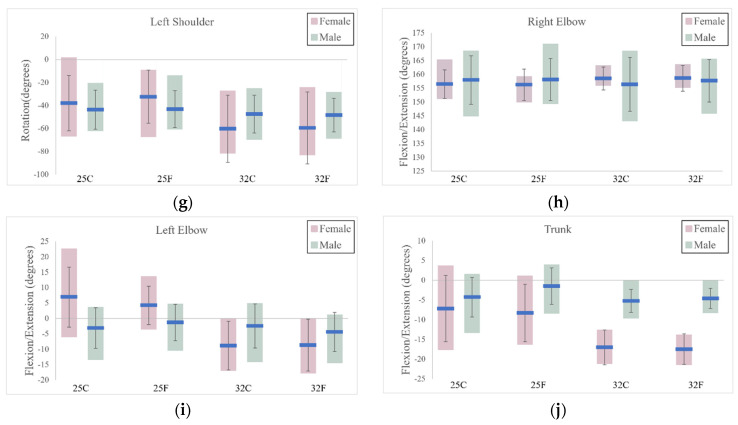
The range of analyzed results within each of the four combinations. (**a**) Compressive force; (**b**) right shoulder_F/E; (**c**) right shoulder_Abd/Add; (**d**) right shoulder_Rot; (**e**) left shoulder_F/E; (**f**) left shoulder_Abd/Add; (**g**) left shoulder_Rot; (**h**) right elbow_F/E; (**i**) left elbow_F/E; (**j**) trunk_F/E.

In Figure 4, each bar represents the range between the minimum and maximum values for males or females within each of the four combinations. Additionally, the average value and standard deviation are included for clarity.

In the statistical analysis, a notable difference between genders was observed for left shoulder abduction/adduction when participants drew the 25 lb bow and aimed at targets at two distances (25C: *p* = 0.048; 25F: *p* = 0.045). Furthermore, significant differences were identified in right shoulder flexion/extension and trunk flexion/extension when using the 32 lb bow at two distances (R_SH_F/E: 32C: *p* = 0.024; 32F: *p* = 0.032; Trunk: 32C: *p* = 0.001; 32F: *p* = 0.0002).

When comparing the 25 lb and 32 lb bows, a significant difference in left shoulder flexion/extension was observed (*p* = 0.049), specifically for females when the target was at a distance of 15 yards. Additionally, for left elbow flexion/extension, *p* values of 0.046 and 0.034 were noted for females when the targets were positioned at distances of 10 yards and 15 yards, respectively.

In the analysis of correlation coefficients, the relationship between compressive force and trunk flexion/extension strengthened as the bow draw weight increased from 25 lbs (r = 0.30) to 32 lbs (r = 0.45) for all participants. Furthermore, a moderate correlation was observed between left elbow flexion/extension and trunk flexion/extension, with an r-value of 0.44 when participants drew the 32 lb bow.

## 4. Discussion

This study examined the forces applied to the lower back when participants utilized two bows with varying draw weights at distinct shooting distances. Additionally, an analysis of corresponding postures was conducted to enhance injury prevention measures and skill development.

When comparing the forces exerted on the lower back between genders, it was observed that males exhibited approximately 100 N more force than females at both shooting distances when using a 25 lb bow. However, this difference increased to over 250 N when switching to a 32 lb bow. Although the exerted forces on the lower back remained below the recommended safety standard of 3400 N [27], it is noteworthy that athletic bows can have a draw weight of up to 60 lbs [28]. A higher draw weight, combined with potentially awkward postures, may increase the risk of injury [29]. One contributing factor to the gender-based difference in lower back forces may be the trunk angle, with females displaying greater trunk extension than males. Notably, as participants tend to flex their trunks, there is a substantial increase in compressive force. This phenomenon may account for the greater standard deviation (SD) observed in males compared with females in Figure 4a. Previous studies [30,31,32] have suggested that reducing trunk flexion can help mitigate the forces exerted on the lower back. Additionally, the correlation between the exposed lower back force and the adopted trunk posture, as indicated by increasing r values with the rise in bow draw weight from 25 lbs to 32 lbs, affirms the relationship between these two variables.

When comparing trunk flexion/extension between genders, variations in adopted shoulder and elbow joint angles may contribute to distinct trunk postures. Notably, a significant difference in the flexion/extension of the right shoulder was observed between genders when pulling the 32 lb bow. This discrepancy is influenced by the variance in muscle strength between males and females [33], leading females to fully extend their right arm to draw the string of the 32 lb bow. This results in a reduced distance between the bow and the upper body for females, especially with increasing draw weight. To accomplish the shooting task with a higher draw weight, females exert more effort on their right upper limb, leading to a smaller right shoulder flexion compared with males. However, when the female participants used a lighter draw weight bow, they could easily draw the bowstring, and may ignore the need for full extension of the right arm. This absence of full extension caused a significant difference in right shoulder flexion for females between the two bows.

An intriguing observation emerges when considering the SD of the right shoulder in females, which was notably lower than that recorded in males during the bow pull with a draw weight of 32 lbs. This discrepancy is attributed to differences in muscle strength, which limits the range of motion for females in fully pulling the bowstring. Conversely, the SD of the left shoulder, specifically in both adduction/abduction and rotation directions, exceeded that of males. This can be attributed to a relatively lower level of muscle strength in females, compelling them to employ left shoulder abduction/adduction and rotation to withstand the reaction force applied from the bow handle.

Moreover, as the draw weight increased from 25 lbs to 32 lbs, females adapted by increasing their left shoulder abduction to precisely aim at targets while fully drawing the string to complete the tasks. Additionally, there was a shift in left elbow flexion/extension angles from positive (flexion) to negative (extension) for females. In contrast, males exhibited a relatively small left elbow extension when using both bows, which can be attributed to the gender-based difference in muscle strength. Due to the comparatively lower muscle strength in females, there is a greater demand for left elbow extension to withstand the reaction force applied from the bow handle. Taking these factors into account, the increased left elbow extension, left shoulder abduction, and right shoulder extension among females contribute to a more pronounced trunk extension as they aim at the target.

When comparing the right shoulder rotation between males and females, specifically in the context of using a 32 lb bow, the average rotation angles for males and females were −47.5° and −24.8°, respectively (a negative value indicates external rotation). Furthermore, among females, the left external rotation angle increased significantly by 30° when switching from a 25 lb to a 32 lb bow. Achieving the crucial shooting step, known as the anchor, requires subjects to draw the bowstring back to their cheek and position either the corner of the mouth or the tip of the nose on the string [34]. However, due to the variance in muscle strength between males and females, the motion of females is significantly restricted when attempting to fully draw a bow with a substantial draw weight.

Based on the findings, it is evident that muscle strength training is important. As highlighted in a prior study [35], the trunk flexors and extensors are associated with chronic low back pain. Additionally, performers are advised to choose bows based on their individual muscle capabilities [29]. This approach aids in maintaining proper postures, ensuring that the spine remains straight without any postural sway. Maintaining a natural position of the shoulders is crucial, avoiding excessive extension or forward and backward flexion. Additionally, implementing a targeted training program to strengthen the adductor and extensor muscles can prove beneficial for enhancing archery skills. Given that the shoulder muscle groups likely play a significant role in ensuring accuracy and stability during archery shooting, such training can contribute to improved performance [36]. Furthermore, a prior study proposed that maintaining 90° elevation in the full drawing position could be an optimal posture to meet the demands of archery [37]. Exploring the application of Rapid Upper Limb Assessment (RULA) analysis for the upper extremities in future studies could offer a more profound understanding of injury assessments in relation to shooting performance. In the case of the release arm’s elbow, it should be directed straight away from the target, and the forearm should be parallel to the ground. All these correct postures not only minimize the risk of injury to the lower back [12] and shoulders [10], but also enhance shooting performance.

## 5. Conclusions

In this study, the Xsens motion tracking system and the JACK Siemens ergonomics tool were employed to assess spinal forces and analyze postures during task performance. The distinct postures adopted by males and females contribute to a significant bias in force exerted on the lower back. Through an examination of the correlation between exposed spinal forces and adopted postures, this study offers valuable insights for the development of training programs. These programs aim to enhance performance skills and mitigate the risk of lower back injuries in the context of archery.

## Figures and Tables

**Table 1:** The genders, body height, body weight, BMI, and experience levels for all participants are listed. The term “BH” denotes body height, while “BW” represents body weight. Similarly, the term “BMI” represents body mass index, which is calculated by the weight in kilograms divided by the height in meters squared.

Subjects	Genders	BH (cm)	BW (kg)	BMI (kg/m^2^)	Levels
S1	F	155	60	25.0	Intermediate
S2	M	178	68	21.5	Expert
S3	F	162	49	18.7	Novice
S4	M	170	61	21.1	Intermediate
S5	F	168	68	24.1	Intermediate
S6	M	160	60	23.4	Intermediate
S7	M	167	61	21.9	Novice
S8	M	182	79	23.8	Expert
S9	F	168	59	20.9	Novice
S10	M	178	117	36.9	Expert
S11	F	170	99	34.3	Novice
S12	M	188	82	23.2	Expert
S13	F	152	62	26.8	Novice

**Table 2:** The analyzed spinal forces and the corresponding joint angles at the pose where a high injury risk may exist.

		Comp (N)	R_Shoulder (°)			L_Shoulder (°)			R_Elbow (°)	L_Elbow (°)	Trunk (°)
			F/E	Add/Abd	Rot	F/E	Add/Abd	Rot	F/E	F/E	F/E
25C	AVE_F	558.46	41.69	115.41	−40.99	14.46	74.70	−37.87	156.42	6.95	−7.17
	AVE_M	662.19	45.49	104.67	−44.31	9.23	83.39	−43.63	157.94	−3.17	−4.28
25F	AVE_F	530.92	41.99	109.51	−37.91	9.87	73.88	−32.45	156.23	4.19	−8.29
	AVE_M	631.11	48.19	103.60	−44.90	10.32	82.22	−43.17	158.10	−1.34	−1.46
32C	AVE_F	576.27	30.96	107.44	−27.75	24.41	78.00	−60.24	158.54	−8.86	−17.02
	AVE_M	890.60	46.63	109.87	−48.41	13.11	81.49	−47.43	156.39	−2.49	−5.24
32F	AVE_F	552.05	27.31	109.39	−24.84	28.76	78.37	−59.54	158.56	−8.66	−17.47
	AVE_M	797.03	44.38	108.70	−47.52	13.07	82.57	−48.33	157.69	−4.43	−4.58

## Data Availability

Data are contained within the article.
